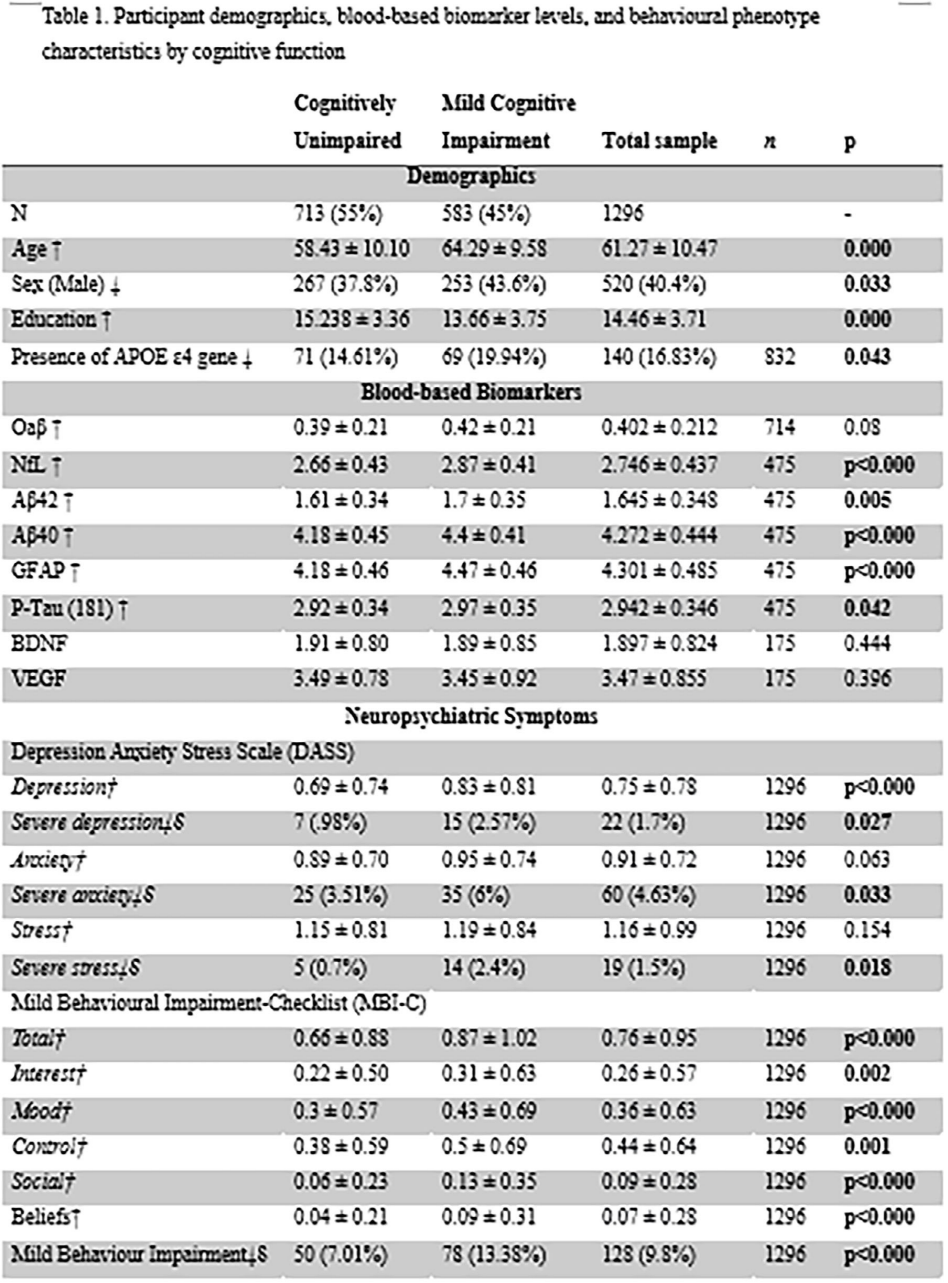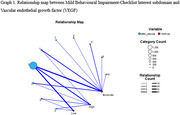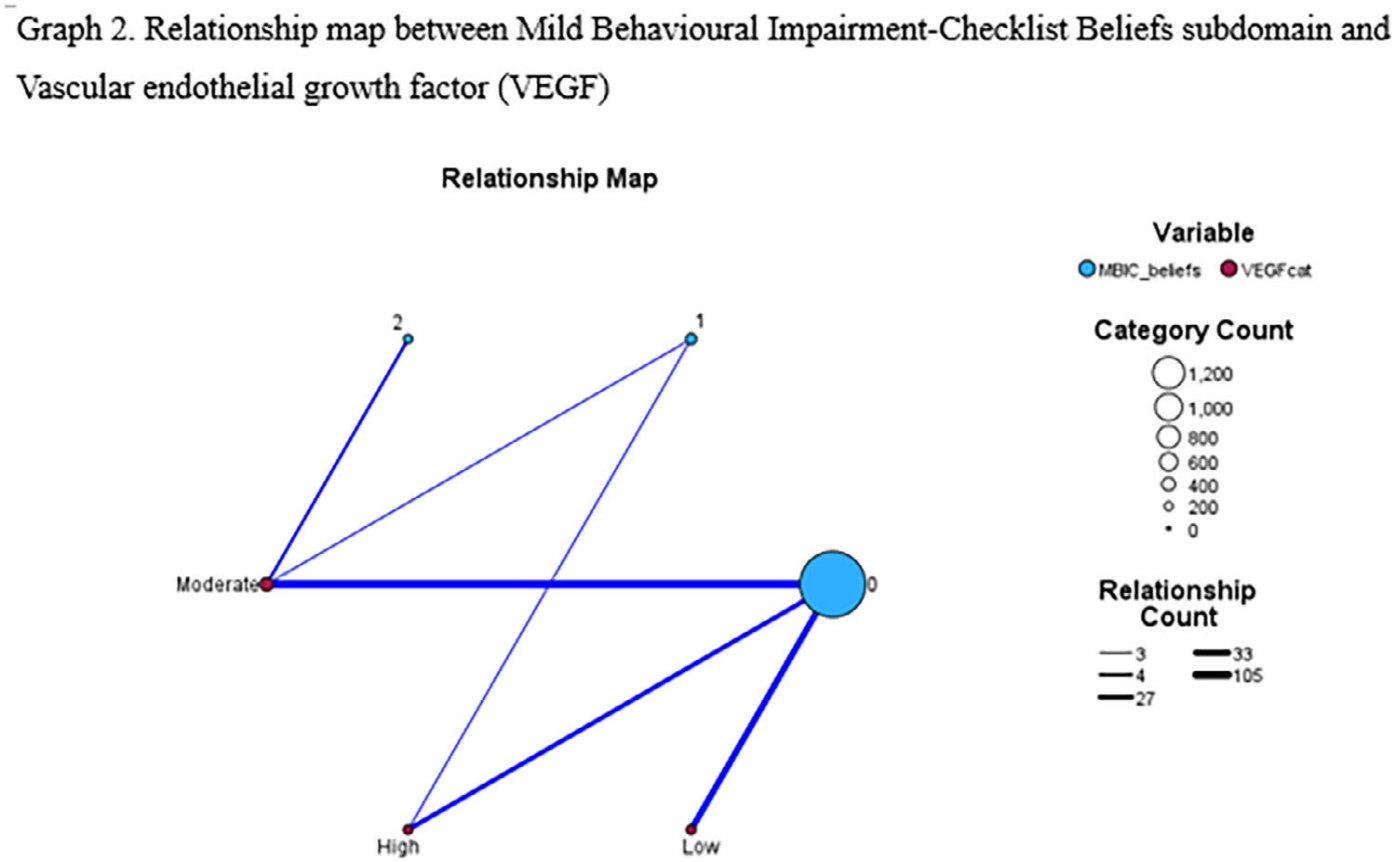# Association between Blood‐based Biomarkers of Alzheimer's disease and Endothelial Function with Mild Behavioral Impairment in a Southeast Asian Population ‐ Biomarkers and Cognition Study, Singapore BIOCIS

**DOI:** 10.1002/alz.092171

**Published:** 2025-01-09

**Authors:** Yi Jin Leow, Gurveen Kaur Sandhu, Ashwati Vipin, Pricilia Tanoto, Mohammed Adnan Azam, Fatin Zahra Zailan, Faith Phemie Hui En Lee, Smriti Ghildiyal, Shan Yao Liew, Isabelle Yu Zhen Tan, Nagaendran Kandiah

**Affiliations:** ^1^ Lee Kong Chian School of Medicine, Nanyang Technological University, Singapore Singapore

## Abstract

**Background:**

Behavioural changes are among the first symptoms noticeable to the person themselves as they begin to experience cognitive decline. Blood‐based biomarkers could potentially be a less invasive and easily detectable biomarker for early identification of Alzheimer's disease and dementia. However, the contributions and pathobiology of blood biomarkers to disease trajectory and prevalence in Asians requires further definition. We examined the association between blood‐based markers(BBM) of Alzheimer's disease and neuropsychiatric symptoms(NPS) in a multi‐ethnic Southeast Asian population.

**Method:**

1296 participants were recruited from the community living in Singapore. 713 were cognitively unimpaired (CU), 583 with mild cognitive impairment (MCI), and overall, the mean age=61.27 years, mean education=14.46years with 39.8% males. Participants underwent blood biomarker tests of Apolipoprotein (APOE) ε4, Aβ oligomers (Oaβ), Neurofilament Light Chain (NfL), Amyloid Peptides(Aβ)42, Aβ40, Phosphorylated Tau at position 181(P‐Tau181), Brain‐derived neurotrophic factor(BDNF), Vascular endothelial growth factor(VEGF) as well as self‐reported questionnaires using the Depression Anxiety Stress Scales(DASS) and Mild Behavioural Impairment‐Checklist(MBI‐C).

**Result:**

In the MCI group, Aβ42(estimate‐0.55; 95% CI[‐0.95, ‐0.15]; p=0.01), Aβ40 (estimate ‐0.361; 95% CI[‐0.72, ‐0.01]; p=0.04), pTau181 (estimate ‐0.484; 95% CI[0.93, ‐0.04]; p=0.03), were significantly associated with overall MBI‐C. Furthermore, Aβ42 was associated with MBI‐C‐interest (estimate ‐0.341; 95% CI[‐0.568, ‐0.114]; p=0.03), and MBI‐C‐control (estimate ‐0.359; 95% CI[‐0.632, ‐0.086]; p=0.01), Aβ40 was associated with MBI‐C‐interest (estimate ‐0.279; 95% CI[‐0.475, ‐0.082]; p=0.005) and MBI‐C‐beliefs (estimate ‐0.077; 95% CI[‐0.153, ‐0.001]; p=0.046), pTau181 was associated with MBI‐C‐social (estimate ‐0.177; 95% CI[‐0.311, ‐0.043]; p=0.01), and BDNF was associated with stress (estimate ‐0.233; 95% CI[‐0.432, ‐0.034]; p=0.022). Interestingly, VEGF was significantly associated with MBIC‐interest (estimate 0.12; 95% CI[0.000, 0.239]; p=0.05) and MBIC‐beliefs (estimate 0.039; 95% CI[0.001,0.078]; p= 0.044).

**Conclusion:**

BBM and NPS were elevated in the MCI group compared to CU. Aβ42, Aβ40, pTau181, NfL, BDNF and VEGF were associated with NPS in community‐dwelling individuals free of dementia. Impairments in neurovascular regulation can lead to neurological and psychiatric symptoms. VEGF might serve as a biomarker for certain neuropsychiatric conditions.